# *Batrachochytrium dendrobatidis* infection in amphibians predates first known epizootic in Costa Rica

**DOI:** 10.1371/journal.pone.0208969

**Published:** 2019-12-10

**Authors:** Marina E. De León, Héctor Zumbado-Ulate, Adrián García-Rodríguez, Gilbert Alvarado, Hasan Sulaeman, Federico Bolaños, Vance T. Vredenburg

**Affiliations:** 1 Department of Microbiology and Molecular genetics, University of California, Davis, United States of America; 2 Department of Biological Sciences, Purdue University, West Lafayette, IN, United States of America; 3 Escuela de Biología, Universidad de Costa Rica, San Pedro, Costa Rica; 4 Departamento de Zoología, Instituto de Biología, Universidad Nacional Autónoma de México, Mexico City, Mexico; 5 Faculdade de Medicina Veterinária e Zootecnia, Universidade de São Paulo, São Paulo, Brazil; 6 Department of Biology, San Francisco State University, San Francisco, California, United States of America; 7 Museum of Vertebrate Zoology, University of California Berkeley, Berkeley, California, United States of America; Vanderbilt University School of Medicine, UNITED STATES

## Abstract

Emerging infectious diseases are a growing threat to biodiversity worldwide. Outbreaks of the infectious disease chytridiomycosis, caused by the fungal pathogen *Batrachochytrium dendrobatidis* (*Bd*), are implicated in the decline and extinction of numerous amphibian species. In Costa Rica, a major decline event occurred in 1987, more than two decades before this pathogen was discovered. The loss of many species in Costa Rica is assumed to be due to *Bd*-epizootics, but there are few studies that provide data from amphibians in the time leading up to the proposed epizootics. In this study, we provide new data on *Bd* infection rates of amphibians collected throughout Costa Rica, in the decades prior to the epizootics. We used a quantitative PCR assay to test for *Bd* presence in 1016 anuran museum specimens collected throughout Costa Rica. The earliest specimen that tested positive for *Bd* was collected in 1964. Across all time periods, we found an overall infection rate (defined as the proportion of *Bd*-positive individuals) of 4%. The number of infected individuals remained relatively low across all species tested and the range of *Bd-*positive specimens was shown to be geographically constrained up until the 1980s; when epizootics are hypothesized to have occurred. After that time, infection rate increased three-fold, and the range of specimens tested positive for *Bd* increased, with *Bd*-positive specimens collected across the entire country. Our results suggest that *Bd* dynamics in Costa Rica are more complicated than previously thought. The discovery of *Bd*’s presence in the country preceding massive declines leads to a number of different hypotheses: 1) *Bd* invaded Costa Rica earlier than previously known, and spread more slowly than previously reported; 2) *Bd* invaded multiple times and faded out; 3) an endemic *Bd* lineage existed; 4) an earlier *Bd* lineage evolved into the current *Bd* lineage or hybridized with an invasive lineage; or 5) an earlier *Bd* lineage went extinct and a new invasion event occurred causing epizootics. To help visualize areas where future studies should take place, we provide a *Bd* habitat suitability model trained with local data. Studies that provide information on genetic lineages of *Bd* are needed to determine the most plausible spatial-temporal, host-pathogen dynamics that could best explain the epizootics resulting in amphibian declines in Costa Rica and throughout Central America.

## Introduction

Amphibians are experiencing a global extinction event [1,2]. Though many factors contribute to population declines, the emergence of the fungal pathogen *Batrachochytrium dendrobatidis* (*Bd*) is one of the most important [3]. The disease chytridiomycosis, caused by the fungal pathogen *Batrachochytrium dendrobatidis* (hereafter *Bd*), was first described in 1999 and has since been found all over the world [3–5]. *Bd* is composed of many genetic lineages that vary in virulence and affect host species differently. The panzootic disease is attributed to *Bd*-GPL, a Global Panzootic Lineage of *Bd* associated with *Bd* epizootics and host population collapse [6]. Other lineages of *Bd* have been shown to be less virulent and have been identified in areas lacking epizootics [7]. *Bd* infects the skin of the amphibian and causes hyperkeratosis, the thickening of skin which disrupts the amphibian’s osmotic balance; leading to death by cardiac arrest in highly infected individuals [8,9].

The dynamics of *Bd* and its hosts, including pathogen invasion and the host-pathogen interactions that follow, are still not fully understood. For example, in some areas (e.g. South Korea, Brazil, and South Africa), *Bd* appears to be in an enzootic state with amphibian hosts [10–12], while in others (e.g. western North America [13], Central America and South America) there are repeated examples of epizootics and die offs of hosts. In these areas, *Bd-*GPL is associated with epizootics [14]. South Korea was recently proposed as a region of high *Bd* genetic diversity, with one of the *Bd* lineages identified as exhibiting genetic hallmarks that may be the source of the panzootic *Bd* (*Bd*-GPL) that emerged in the 20^th^ century [15]. Many of the reported declines attributed to *Bd* in the New World such as in southern Mexico, Guatemala, and Costa Rica occurred decades before *Bd* was described, thus, retrospective studies can help create a timeline for *Bd* emergence and spread [16].

Causes of amphibian declines in Costa Rica, where some of the earliest reported declines of amphibians occurred, have been debated in the literature [17,18]. Some studies proposed *Bd* epizootics occurred when environmental factors weakened host immune systems making them more susceptible to endemic *Bd* [19]. Other studies refute this and show that an invasive *Bd* pathogen caused the epizootics [16,20]. Costa Rica had one of the earliest amphibian declines (1980s and 1990s) that was later associated with *Bd* epizootics [21–23]. These declines mostly affected stream-dwelling species at elevations between 1000 and 2500 meters and include sites such as Monteverde, where the amphibian community collapsed a decade before *Bd* was described [24]. At this site, around the year 1987, half of all amphibian species, along with the Costa Rican golden toad (*Incilius periglenes*), disappeared [22].

Like many other areas experiencing *Bd* epizootics, anuran (frogs and toads) species in Costa Rica experienced differential susceptibility to *Bd*. Whether this is due to different immune responses by hosts or possibly exposure to different lineages of *Bd* is not known [25]. For example, all nine frog species within the *Craugastor punctariolus* clade (robber frogs) [26,27] declined across all their elevational range, from 0 to 2300 meters a.s.l. [28], and yet decades later they appear to be slowly recovering from past *Bd* epizootics [25,29,30]. Similar cases of catastrophic decline followed by apparent recovery have been observed in some highland populations of harlequin frogs, tree frogs and ranid frogs [31,32]. Population fluctuations such as these elicit questions regarding the role of *Bd* transmission, virulence, and lineage in this disease system. Recent studies have shown that *Bd-*GPL is unlikely to be endemic to Costa Rica though it is possible that other endemic *Bd* lineages occur in Costa Rica and throughout the Americas [33].

Retrospective studies analyzing the presence of *Bd* in specimens preserved in natural history collections have been useful to describe *Bd* invasions that may have led to amphibian declines [16,34] as well as situations where *Bd* has been present for a century [10,35,36]. Museum collection data has also contributed to tracking and identifying declined species [37–39]. The utilization and analysis of accurate collections and databases is crucial to understanding the historical context of population declines and can result in more applicable conservation plans. We conducted a retrospective survey using a *Bd* qPCR assay effective on museum specimens [16] to describe the spatial and temporal patterns of *Bd* of anurans in Costa Rica from 1961–2011. We used logistic regression analysis to examine possible environmental factors correlated with *Bd* infections. Based on our data, we also constructed a habitat suitability model for *Bd* in Costa Rica using a MaxEnt model in order to visualize *Bd* habitat suitability for the region.

## Materials and methods

### Data collection

This study was approved by the San Francisco State University IACUC (Vredenburg #A16-01). We sampled 1016 formalin-fixed, ethanol-preserved museum specimens including thirty-four species of frogs from five taxonomic families. All specimens were collected in Costa Rica between 1961 and 2011 and are housed in the Museum of Zoology (UC Berkeley) and at the Universidad de Costa Rica (UCR). We focused our sampling efforts on anuran species that were reported to have declined during the 1980s and 1990s [40]; however, museum specimens collected in the past were not intended for disease studies therefore conclusions about the dissemination and presence of *Bd* are inherently limited. Most of the *Craugastor* species have not recovered from population declines and are still classified as critically endangered or extinct according the IUCN [41]. *Craugastor* frogs are direct developers with no aquatic tadpole phase, therefore less dependent on water for reproduction. Because *Bd* is often considered a water-borne pathogen, we questioned whether *Craugastor* declines were associated with *Bd* or if something else was affecting survival. We also chose species with breeding behavior associated with water and which populations initially declined in the 1980s and 1990s and were observed to be recovering by around 2010 or later (*Agalychnis annae*, *Agalychnis lemur*, *Lithobates vibicarius* and *Lithobates warszewitschii*). The data from our skin swabs, including qPCR results from our survey and geographic coordinates for all samples used in this study can be freely accessed on the amphibian disease portal (https://amphibiandisease.org), DOI: <https://n2t.net/ark:/21547/Auf2> [42].

### Quantitative PCR Assay

We collected skin swabs from formalin-fixed frogs following a standardized protocol that reduces chances of cross-contamination between specimens and is described in Cheng et al. (2011) [16]. Each museum specimen was removed from its jar using flame-sterilized forceps and thoroughly rinsed with 70% ethanol to remove any surface contaminants. Flame-sterilized forceps were used to hold the specimen while swabbing five times each of the following locations for a total of twenty-five strokes, using sterile fine rayon-tipped swabs (MW113, MWE, United Kingdom); l) ventral surface from mid abdomen to cloaca, 2) each inner thigh, and 3) the bottom side of the webbing between each digit. Swabs were stored in 1.5mL tubes with tether cap (Fisher USA) and stored at -4˚C until processing. New latex or nitrile gloves were used at all times when handling tubes, jars, and specimens and were changed between handling each specimen. We followed the method detailed in Boyle et al. (2004) and Cheng et al. (2011) for our qPCR assay and we processed all of our samples in the Vredenburg Lab at San Francisco State University [16,43,44]. Before extraction, swabs were put in a spinvac to evaporate any ethanol which could inhibit qPCR. *Bd* was then extracted from swabs using the Prepman Ultra (Thermo Fisher Scientific) and diluted. For qPCR, positive and negative controls (water, TE buffer) were run in triplicate, while a PrepMan negative control were run in singlicate on every 96-well PCR plate to detect possible environmental contamination. Standard curves were calculated using positive controls with known 100, 10, 1, and 0.1 *B*. *dendrobatidis* zoospore genomic equivalents, provided by A. D. Hyatt. Samples were run on an Applied Biosystems 7300 Real-Time PCR thermocycler. All samples were tested in singlicate unless they showed exponential amplification before cycle 50 or amplification was observed in any of the negative control wells. Samples which amplified early or showed non-sigmoidal amplification curves were rune two additional times. We determined those samples as positive if two of three runs showed exponential amplification before cycle 50 [16].

### Statistical analyses

We performed all statistical analyses using the software R (version 3.5.0). To characterize the temporal and spatial dynamics of *Bd* in Costa Rica, we calculated 95% confidence intervals for the proportion of *Bd*-positive individuals for each decade sampled based on a binomial probability distribution (Table 1). We quantified the presence of *Bd* as the number of *Bd*-positive individuals divided by the total number of sampled individuals within a taxonomic unit, geographic area, and/or time frame. We performed a linear regression for the likelihood of an individual to be *Bd*-positive, with *Bd* infection status as a response variable, assuming a binomial distribution as individuals are either infected or non-infected.

**Table 1:** *Batrachochytrium* dendrobatidis (*Bd*) prevalence in museum specimens collected in Costa Rica. Pr (no *Bd*) is the probability of finding no *Bd*-positive samples in each time period if *Bd* were present with an enzootic prevalence of 11.0% [35].

Decade	Tested Negative	Tested Positive	Sample Size	Prevalence (%)	Pr (no *Bd*)
1960–1970	333	15	348	4.31	< 0.001
1971–1980	355	17	372	4.57	< 0.001
1981–1990	151	20	171	11.70	< 0.001
1991–2000	72	9	81	11.11	< 0.001
2001–2011	37	7	44	15.91	0.006

**Fig 1:**
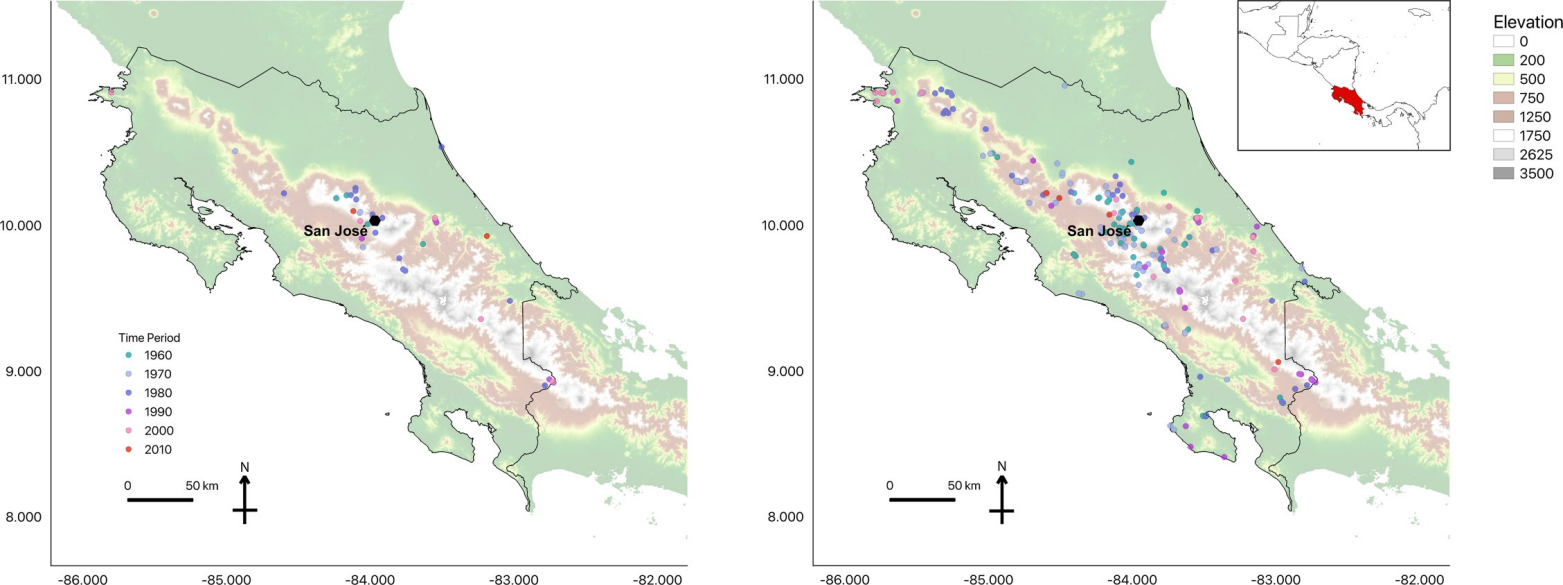
**Amphibian museum specimens (1964–2011) tested positive (a) and negative (b) for *Bd* in Costa Rica (outlined).** Black octagon denotes San José, the capital of Costa Rica.

For the linear regression, we used the elevation and 19 bioclim variables available on WorldClim (http://www.worldclim.org) and reduced the number of variables by performing a Pearson-correlation test to eliminate highly correlated factors (>0.9 or <-0.9). The following variables were used: annual mean temperature, mean diurnal temperature range, day-to-night temperature oscillations relative to the annual oscillations (isothermality), temperature seasonality, annual precipitation, precipitation of the wettest month, precipitation of the warmest quarter, precipitation of the driest quarter, and precipitation of the coldest quarter. We then performed a stepwise regression to choose the best-fit model based on the AIC [45,46]. Fig 1 was generated using QGIS (http://qgis.osgeo.org), using elevation data from USGS (https://www.usgs.gov), and shapefiles from GADM (gadm.org).

Lastly, we used MaxEnt to estimate *Bd* habitat suitability using the variables shown to be significant in predicting the possibility of finding *Bd*-positive individuals from the linear regression model [47].

## Results

**Fig 2:**
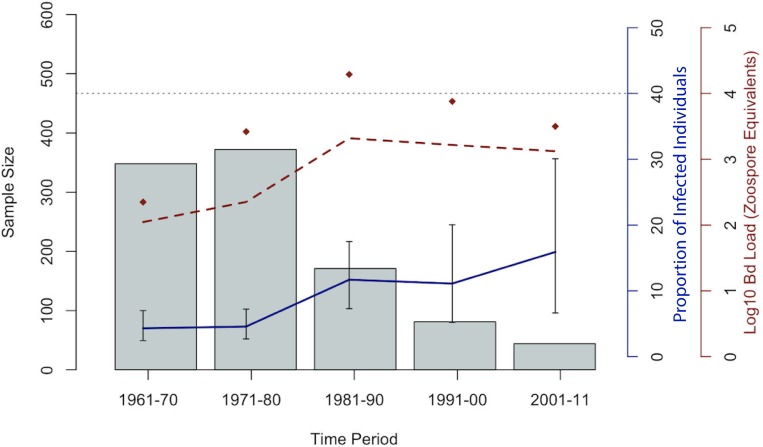
Emergence of *Bd* in Costa Rica from 1961–2011. For samples tested in every decade between 1961–2011, the solid blue line represents the proportion of individuals tested positive for *Bd*. The average infection intensity, measured by the zoospore equivalents detected, is represented by the dashed red line. The maximum zoospore equivalents value is represented by red diamonds and gray bars represent the number of samples analyzed. The horizontal dashed line at Log10(10,000) marks the Vredenburg 10,000 value, the infection load which has been shown to cause mortality in anurans [13].

Our qPCR analysis of the 1016 museum specimens collected between 1961 and 2011 revealed sixty-eight *Bd*-positive anuran samples and 948 *Bd*-negative anurans for an overall *Bd* infection rate of *Bd* 6.7% (n = 1016). The earliest records were detected in four *Lithobates vibicarius* specimens collected in 1964 from the central volcanic mountain range, on the hillsides of Poas Volcano (Fig 1A, S1 Table). UCR museum records of the species included in this study begin in the 1960s, thus our retrospective survey begins with the oldest specimens collected in 1961 (n = 2). When grouped by decades, we find relatively low rate of *Bd* presence in the 1960s and 1970s (4.31% and 4.57%, n = 372 and n = 348; respectively), followed by an increase in the 1980s to 11.70% (n = 171). By the late '80s and '90s, *Bd* was detected in museum specimens collected throughout the entire country, whereas earlier positive samples were obtained only from frogs collected in the central regions of the country. Thus, *Bd* became more common throughout the country in the late 1980s and 1990s (Figs 1A and 2); however, conclusions derived from the presence of *Bd* based on museum specimens that were not collected for disease studies are limited. Overall, the majority of the *Bd* positive samples were found in mountains throughout Costa Rica at elevations ranging from 32–2550 meters a.s.l. We found that the rate of *Bd* detection between species ranged between 0.0% and 46.7%. The species with the highest percentage of *Bd* positive samples came from species that are highly dependent on water for reproduction or live in close proximity to water. For example, we found 46.7% *Bd* infection rate (n = 15) in the stream-breeding frog *Hyloscirtus palmeri*, and 45.9% prevalence (n = 37) in *Lithobates vibicarius*, a highland pond-breeding frog. The lowest level of *Bd* detection occurred in the Dendrobatidae and Bufonidae families, in species that spend much of their time on land rather than in water. However, we sampled only a small number of Dendrobatidae specimens (n = 7), and no samples were *Bd*-positive, whereas in Bufonidae we sampled 171 specimens and found that 0.6% were *Bd*-positive (S1 Table). Overall, the Ranidae family showed the highestpresence of *Bd* (22.4%; n = 300), followed by Craugastoridae (7.4%; n = 453). Most Craugastoridae samples were taken from direct developing streamside-breeding species of the *Craugastor punctariolus* clade, which are critically endangered across their entire distribution. *Craugastor andi* however, a species not categorized within the *punctariolus* clade, is also found near streams [28]. In the Craugastoridae family, only four *C*. *escoces* individuals tested positive out of sixty-three specimens. All *C*. *escoces* samples were collected before the 1987 Costa Rican amphibian population decline epizootic [24]. The earliest *Bd*-positive *C*. *escoces* specimens were collected in 1975, and the last was collected in 1986.

**Fig 3:**
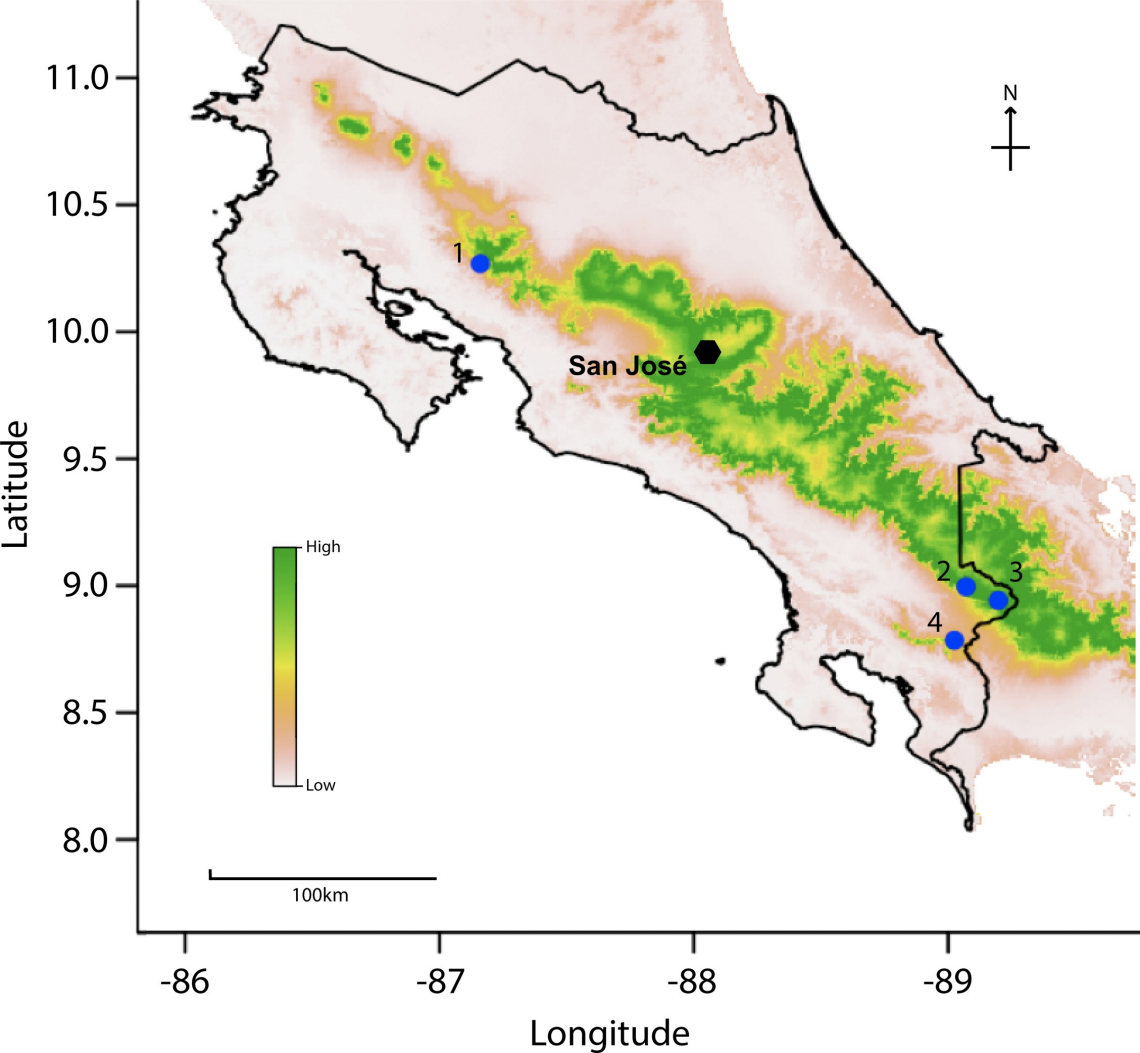
*Bd* habitat suitability model. Areas in Costa Rica predicted to have *Bd* suitability. Increased intensity from white to green indicates increased suitability while blue dots are *Bd*-epizootic localities. (1) Monteverde, where the 1987 decline occurred and (2–4) indicates declines between 1993–1994.

Our power analysis showed that we had enough samples within each time period to have a robust statistical test (p < 0.01 across all time periods sampled; Table 1). In the model with the best AIC (AIC = -2853.44), we found that infection status has a positive relationship with elevation and annual mean temperature (p<0.001 and p<0.001; respectively). We also found that *Bd* infection status has a negative relationship with mean diurnal temperature range (p<0.001). Infection status was not shown to have a significant relationship with the following factors: isothermality, precipitation of the warmest quarter, precipitation of the coldest quarter, precipitation of the wettest month, annual precipitation, temperature seasonality, and precipitation of the driest quarter (Table 2). Areas predicted by the *Bd* habitat suitability model to be suitable for *Bd* includes mid-elevation ranges across central Costa Rica (Fig 3). The areas predicted to be unsuitable include the lowland regions and the coasts.

**Table 2:** Linear regression output. Environmental factors and their relationship to *Bd* infection status. (+) next to the variable name indicates a positive relationship between factor and *Bd* presence. (-) indicates a negative relationship between factor and *Bd* presence. The model with the lowest AIC value (1st model; AIC = -2853.44) was considered the best model.

Variables	Model 1	Model 2	Model 3	Model 4
Elevation (+)	X	X	X	X
Annual Mean Temperature (+)	X	X	X	X
Mean Diurnal Temperature Range (-)	X	X	X	X
Isothermality	X	X	X	X
Precipitation of Wettest Month	X	X	X	X
Precipitation of Warmest Quarter	X	X	X	X
Precipitation of Coldest Quarter	X	X	X	X
Annual Precipitation		X	X	X
Temperature Seasonality			X	X
Precipitation of Driest Quarter				X
AIC	-2853.44	-2851.88	-2849.93	-2847.93
Δ AIC	1.56	1.95	2.00	N/A

## Discussion

Our retrospective study using museum specimens revealed that *Bd* was present in Costa Rica at least two decades before declines were discovered at Monteverde [20], and four decades before *Bd* was described. It is possible that the DNA of old samples are more degraded than recent samples, which could lead to a spurious signal of emergence. However, several retrospective studies of *Bd* infections in amphibians have shown consistent and relatively high *Bd* detection rate (20–40%) for specimens over an entire 100-year period [35,48].

We found an increase in *Bd* detection throughout Costa Rica beginning in the 1970s (Fig 1A), with detection rate escalating during and after the decade of the first known epizootic (1980s), which supports the pattern expected with an invasive pathogen. Here, we show a spatial pattern where *Bd* appears to be present at a low detection rate across some of the region (Fig 1A), before purported enigmatic declines occurred. This could be indicative of an invasive pathogen that invades unsuccessfully for decades before epizootics develop, or it could be that the earlier *Bd* infections were the result of a non-virulent lineage of *Bd*, such as has been identified in other parts of the world [35,49,50]. Consistent with an invading pathogen, our limited data show a pattern of spread across the entire time of the study. We found *Bd*-positive specimens only in and around central Costa Rica in the earlier time period (1960s), but by the 1970s, we found *Bd*-positive individuals across a larger area, from northwestern areas to southern areas of Costa Rica. Our results from the 1980s show the greatest expansion of *Bd*, with *Bd*-positive individuals found on the eastern coast of Costa Rica and near the Panama-Costa Rica border to the southeast and all the way north close to Nicaragua. This pattern might reflect the *Bd*-epizootics that are proposed from that time period. In the more recent decades (e.g. 1990s), there were fewer specimens in museum collections that we could test. Thus, constructing a robust statement regarding the spatial distribution of *Bd* in the more recent time period is not possible given the available specimens. Our samples are not free from sampling biases, since museum specimens were collected for reasons unrelated to our study.

In some areas (e.g. South Korea, Brazil, and South Africa), where both the Global Panzootic Lineage (GPL) and an endemic strain of *Bd* occur in sympatry, direct competition between pathogen strains and potential cross immunity of hosts may explain the lack of epizootics [11,51]. Our study provides evidence that *Bd* was present in Costa Rica before the 1987 epizootic in Monteverde, but we acknowledge that there may have been previous undetected epizootics especially since the pathogen was yet to be described. The few specimens collected in the 1970s showed relatively high levels of infection and, by the 1980s, both *Bd* infection rate and average zoospore equivalents increased (Figs 1A and 2). Our data do not refute the studies that show that epizootics in Central America are associated with *Bd* invasion, but they do help provide further data for interpretation. For example, identifying the *Bd* lineage of our earlier positive *Bd* samples would be extremely helpful, since having multiple pathogens that are closely related to each other in a population of hosts may help us understand why *Bd* has had such variable effects on hosts, even in areas with epizootics. Sub-lethal effects from fighting off the infection of one pathogen can suppress host immunity against other stressors, causing a larger effect [52–54]. However, populations can also benefit by being exposed to a lower virulence pathogen before being exposed to a similar yet more virulent pathogen. Direct competition between pathogens and/or cross immunity has been shown to assist the hosts in acquiring partial or total immunity to one pathogen from a previous infection by another closely related pathogen [55]. Additionally, climate change and the stress of an inconsistent environment may negatively affect amphibians and result in suppressed immune systems, which could make amphibians more vulnerable to chytridiomycosis [56–58]. Future studies involving *Bd* genotyping are required to determine whether *Bd* found in Costa Rica are of a single lineage and whether or not the *Bd* found in epizootics and enzootics are of the same lineage.

Consistent with other studies, our linear regression results found that *Bd* infection status has a positive relationship with elevation and mean temperature [59,60], but contrary to other studies, our best linear regression model (Table 2), did not show a relationship between precipitation and *Bd* detection rate [61–64]. This may be due to unintended sampling bias. For example, frogs in the genus *Craugastor* made up a large proportion of available specimens (434 of 1016) and yet most were negative. Frogs in the genus *Craugastor* develop directly from terrestrial eggs. This more terrestrial lifestyle may decrease exposure to the aquatic pathogen *Bd*, although terrestrial life history alone is not associated with susceptibility to infection [65,66].

Our data shows a higher detection rate of *Bd* in mid to high elevation species, possibly due to a more suitable climate for *Bd* [67–69]. The *Bd* habitat suitability model (*Bd* HSM) we produced which is specific to Costa Rica, predicts high habitat suitability for *Bd* where epizootics occurred (Fig 3), and is similar to previous *Bd* HSM studies [64,65].

Our model also identifies high elevations along the central mountain range as having the highest *Bd* suitability and should be prioritized for further research and monitoring. The zoospore equivalents (i.e. the Zswab, a measure of infection intensity or host infection load) and detection of *Bd* observed in this study are typically consistent with enzootic dynamics [66], and unfortunately no samples were collected that would allow for a description of host/pathogen dynamics in populations of hosts [13]. Of the *Bd*-positive samples, none of the individuals showed observable symptoms of disease at the time of swabbing for *Bd*. Many of these specimens were collected before *Bd* was described and were not collected for disease studies. Collectors do not typically select animals that look sick as representatives of their collections. Our study utilizes qPCR to detect *Bd*, and we were able to detect specimens with low zoospore equivalents. Quantitative PCR was found to be more sensitive and less invasive than histological assays [Cheng et al. 2011]. Performing histology on otherwise healthy looking specimens would require destructive sampling of museum specimens and is beyond the scope of this study. Nonetheless, our results add important information that may help in our understanding of the mass die offs of amphibians that occurred in Costa Rica. Our results show that although *Bd* was present in the 1960s, the significant increase in *Bd*-positive individuals did not begin in the samples available until the 1980s (when the epizootics began). The data we provide in this study are not well-suited to test the novel vs endemic pathogen hypotheses for *Bd* (Fig 3) [18]; however, these samples could be used to test for *Bd* lineage in future studies that may clarify this question [49]. The steady increase in *Bd* presence throughout all elevations in Costa Rica after 1990 suggests that *Bd* has become more broadly established throughout the country [25,70] than it was previously. The recent rediscovery of some remnant populations of frogs once thought extinct provides new opportunities to assess the current impact of *Bd* in highly susceptible species [25,29,30,32]. Consistent with previous studies, we propose that *Bd* epizootics in amphibians may have begun in the central range of Costa Rica, affecting stream-breeding and pond-breeding species that inhabited this region (S1 Table and 2) such as *Lithobates vibicarius*, *Isthmohyla angustilineata*, *I*. *tica*, *I*. *xanthosticta*, *I*. *rivularis*, *Duellmanohyla uranochroa Craugastor fleischmanni*, *C*. *ranoides*, *C*. *escoces*, *C*. *sp*. (*C*. *punctariolus* clade), *C*. *melanostictus*, *C*. *andi*, *Atelopus varius*, *A*. *senex* (Harlequin frogs), and *Incilus holdrigei*.

Disease ecology research using museum specimens allows for a retrospective view. This can be extremely valuable as with the case of the global pandemic in amphibians caused by *Bd* [35,71,72]. Most of the declines attributed to *Bd* epizootics (including in Costa Rica) occurred before *Bd* was described (1999) [16,36,73]. In this study, we discovered *Bd*-infected frogs in Costa Rica twenty-three years before enigmatic amphibian declines occurred and 34 years before *Bd* was discovered [17]. The infected animals in this study could indicate the occurrence of any one or multiple epidemiological dynamics including: failed pathogen invasions (e.g. “pathogen fade out” theory [74,75]), slower than expected invasion dynamics resulting in epizootics, endemic lineages of *Bd* that may exhibit enzootic pathogen/host dynamics, an extinct endemic *Bd* lineage; or an earlier *Bd* lineage that evolved or hybridized into the current *Bd* lineage. Additional studies that sequence and identify *Bd* lineages from our data will help create a more complete understanding of *Bd* phylogenetics in Central America.

## Supporting information

S1 Table *Bd* observed in in museum specimens from Costa Rica. The table shows surveyed species, conservation status and proportion of samples with *Bd* including 95% binomial confidence intervals.
(DOCX)
