# Supplementary material for: Batrachochytrium dendrobatidis infection in amphibians predates first known epizootic in Costa Rica
Source: PLoS One. 2019 Dec 10;14(12):e0208969. doi: 10.1371/journal.pone.0208969 (PMC6903748; doi:10.1371/journal.pone.0208969)
Supplement: S1 Table — The table shows surveyed species, conservation status and proportion of samples with Bd including 95% binomial confidence intervals. (DOCX) [file pone.0208969.s001.docx]

**S1 Table.** *Bd* observed in in museum specimens from Costa Rica. The table shows surveyed species, conservation status and proportion of samples with *Bd* including 95% binomial confidence intervals.

| **Family** | **Genus species** | **IUCN Red List** | **Total #**  **(# positive)** | **% Positive**  **(95% CI)** |
| --- | --- | --- | --- | --- |
| **Bufonidae** |  |  | **171 (1)** | **0.6 (0.0-3.2)** |
|  | *Atelopus chiriquiensis* | CR | 39 (0) | -- |
|  | *Atelopus senex* | CR | 30 (0) | -- |
|  | *Atelopus varius* | CR | 30 (0) | -- |
|  | *Incilius fastidiosus* | CR | 19 (1) | 5.3 (0.1-26.1) |
|  | *Incilius holdridgei* | CR | 41 (0) | -- |
|  | *Incilius periglenes* | EX | 12 (0) | -- |
| **Craugastoridae** |  |  | **453 (33)** | **7.3 (5.1-10.1)** |
|  | *Craugastor andi* | CR | 20 (1) | 5 (0.1-24.9) |
|  | *Craugastor angelicus* | CR | 57 (1) | 1.8 (0.0-9.4) |
|  | *Craugastor catalinae* | CR | 1 (1) | 100 (2.5-100) |
|  | *Craugastor escoces* | EX (rediscovered) | 63 (4) | 6.4 (1.8-15.5) |
|  | *Craugastor fleischmanni* | CR | 92 (9) | 9.8 (4.6-17.8) |
|  | *Craugastor melanostictus* | LC | 25 (5) | 20 (6.8-40.7) |
|  | *Craugastor obesus* | EN | 5 (1) | 20 (0.1-71.6) |
|  | *Craugastor ranoides* | CR | 98 (5) | 5.1 (1.7-11.5) |
|  | *Craugastor rhyacobatrachus* | EN | 6 (1) | 16.7 (0.4-64.1) |
|  | *Craugastor taurus* | CR | 31 (0) | -- |
|  | *Craugastor sp.* | CR^*^ | 36 (4) | 11.1 (3.1-26.1) |
|  | *Pristimantis caryophyllaceus* | NT | 20 (1) | 5 (0.1-24.9) |
| **Dendrobatidae** |  |  | **7 (0)** | -- |
|  | *Silverstoneia nubicola* | NT | 7 (0) | -- |
| **Hylidae** |  |  | **300 (15)** | **5.0 (2.8-8.1)** |
|  | *Agalychnis annae* | EN | 47 (2) | 4.3 (0.5-14.5) |
|  | *Agalychnis lemur* | CR | 54 (0) | -- |
|  | *Duellmanohyla rufioculis* | LC | 32 (0) | -- |
|  | *Duellmanohyla uranochroa* | EN | 19 (0) | -- |
|  | *Hyloscirtus colymba* | CR | 5 (0) | -- |
|  | *Hyloscirtus palmeri* | LC | 15 (7) | 46.7 (21.3-73-4) |
|  | Isthmohyla angustilineata | CR | 12 (1) | 8.3 (2.1-38.5) |
|  | *Isthmohyla calypso* | CR | 1 (1) | 100 (2.5-100) |
|  | *Isthmohyla pictipes* | EN | 37 (2) | 5.4 (0.66-18.19) |
|  | *Isthmohyla rivularis* | CR | 40 (2) | (0.61-16.92) |
|  | *Isthmohyla tica* | CR | 26 (0) | -- |
|  | *Isthmohyla xanthosticta* | DD | 1 (0) | -- |
|  | *Ptychohyla legleri* | EN | 11 (0) | -- |
| **Ranidae** |  |  | **85 (19)** | **22.4 (14.1-32.7)** |
|  | *Lithobates vibicarius* | CR | 37 (17) | 48.7 (32.4-65.2) |
|  | *Lithobates warszewitschii* | LC | 48 (2) | 4.2 (0.5-14.3) |
| **Total** | **34** |  | **1016 (68)** | **6.7 (5.2-8.4)** |

EX= extinct, CR= critically endangered, EN= endangered, NT= near threatened, LC= least concern, DD= data deficient, *= this population.
